# *Caenorhabditis elegans* as an experimental model for resilience-boosting microbiota interventions in nonmodel ectotherms

**DOI:** 10.1093/femsre/fuag031

**Published:** 2026-07-10

**Authors:** Charlotte Sprason, Philip Donkersley, Jason P Chin, Alexandre Benedetto

**Affiliations:** Biomedical and Life Sciences, Lancaster University, Bailrigg Lancaster, LA1 4YW, United Kingdom; Lancaster Environment Centre, Lancaster University, Bailrigg Lancaster, LA1 4YW,United Kingdom; School of Biological Sciences, Queen’s University Belfast, Belfast19 Chlorine Gardens, BT9 5DL, United Kingdom; Lancaster Environment Centre, Lancaster University, Bailrigg Lancaster, LA1 4YW,United Kingdom; School of Biological Sciences, Queen’s University Belfast, Belfast19 Chlorine Gardens, BT9 5DL, United Kingdom; Biomedical and Life Sciences, Lancaster University, Bailrigg Lancaster, LA1 4YW, United Kingdom

**Keywords:** ectotherm, microbiota, microbiome, *Caenorhabditis elegans*, stress, environment

## Abstract

Ectotherms make up most animal species and deliver essential services to ecosystems and human food supply chains but are highly vulnerable to environmental perturbations. Being vastly underrepresented in laboratory-based studies, we also critically lack knowledge and approaches to improve their resilience to emerging threats from climate change and anthropogenic activities. With the gut microbiota critically modulating animal health, including stress tolerance, gut microbial interventions may offer opportunities to improve nonmodel ectotherm resilience to such pressures. Developing such interventions requires knowledge of host–gut microbiota–environment interactions that is critically lacking for most species and needs establishing. The roundworm *Caenorhabditis elegans* is emerging as a powerful ectotherm model to study host–gut microbiota interactions. Extensively utilized across research fields, *C. elegans* offers a breadth of resources and methodologies, including bioengineering technologies, defined microbiotas, and host and microbial isolate collections. In this review, we recapitulate *C. elegans* uses in gut microbiota studies, highlighting strengths and limitations and how it may accelerate mechanistic study of nonmodel ectotherm microbiotas. We notably propose a *C. elegans*-based strategy to identify, isolate, and study isolates from nonmodel ectotherm species to design microbial interventions that may promote climate resilience in nonmodel species.

## Teaser

Interventions into the gut microbiotas of nonmodel ectotherms could improve their resilience to environmental stressors but said microbiotas are under-investigated: *Caenorhabditis elegans* models can help close the gap.

## Introduction

Comprising over 99% of all known multicellular eukaryotic species (Atkinson and Sibly 1997), ectotherms are animals that have limited internal control of their body temperature. Since they depend on the environment for temperature regulation, ectotherms are at particular risk from climate change. Changes such as increased average and extreme temperatures, as well as daily fluctuations in temperature (Paaijmans et al. 2013), and moisture levels (Rohr and Palmer 2013) have been implicated in population declines seen across terrestrial and aquatic vertebrate and invertebrate ectotherms (Weinbach et al. 2018, Lachs et al. 2021, Doucette et al. 2023, Hayden Bofill and Blom 2024). Anthropogenic activities on the environment also directly influence populations. For example, wild bee populations have been declining globally, suffering an estimated 25% loss in species diversity between the pre-1990 and the 2006–2015 periods (Zattara and Aizen 2021), while in eastern England, a third of pollinator species have declined between 1980 and 2013 (BugLife and WWF 2019).

If such trends continue, the disappearance of ectotherm populations, whether cnidarians, arthropods, fish, amphibians, or reptiles could have extensive effects, from local ecologies to global economies (Perrings et al. 1992, Cardinale et al. 2012). Within their native environments, insects cycle nutrients, pollinate plants, disperse seeds, maintain soil structure and fertility, control populations of other organisms, and provide a major food source for higher taxa (Scudder 2017). Accordingly, the global pollination benefit was predicted to be worth between US$235 and $577 billion in 2010 (Lautenbach et al. 2012). Amphibians and reptiles have extensive ecological roles as keystone species in their environments, within food webs, acting as sentinel species, and performing pest control, environmental cleanup, pollination, and seed dispersal (Cortes et al. 2014). In aquatic environments, corals act as a diverse habitat, providing coastal protection, and supporting a wide range of life (Veron et al. 2009), which in turn supports the fish and seafood market valued at US$676.10 billion in 2024 (Statista 2024). Loss of fish populations, notably from overfishing, has been shown to lead to a variety of economic and environmental complications (Srinivasan et al. 2010, Du et al. 2021). Hence, it is important to devise and implement strategies to protect ectotherms, and the industries that rely on them, from both climate change and damaging human activities. While current corrective approaches revolve around adopting more sustainable practices (Boström-Einarsson et al. 2020, Hayden Bofill and Blom 2024, Sinervo et al. 2024, Sanz-Martín et al. 2026), new complementary and faster-acting strategies may be possible.

The microbiome is the community of symbiotic (commensal, mutualistic, or pathogenic) microorganisms that live on or within a larger host organism (Lederberg and McCray 2001). A microbiota is the assemblage of living microorganisms present exclusively in a defined host environment, such as an animal’s lower gut or skin (Marchesi and Ravel 2015). Microbiome and microbiota research has garnered increasing interest for the past 20 years, for its potential to develop health interventions in key animal and plant species (Shreiner et al. 2015, Ali et al. 2023, Miller et al. 2024). In particular, ectotherm gut microbiotas [such as those of fence lizards; (Moeller et al. 2020), fruit flies (Jaramillo and Castañeda 2021), and tilapia (Wu et al. 2025)] have been found to modulate their hosts’ response to temperature, including increasing their heat tolerance (Fontaine and Kohl 2023).

**Figure 1:**
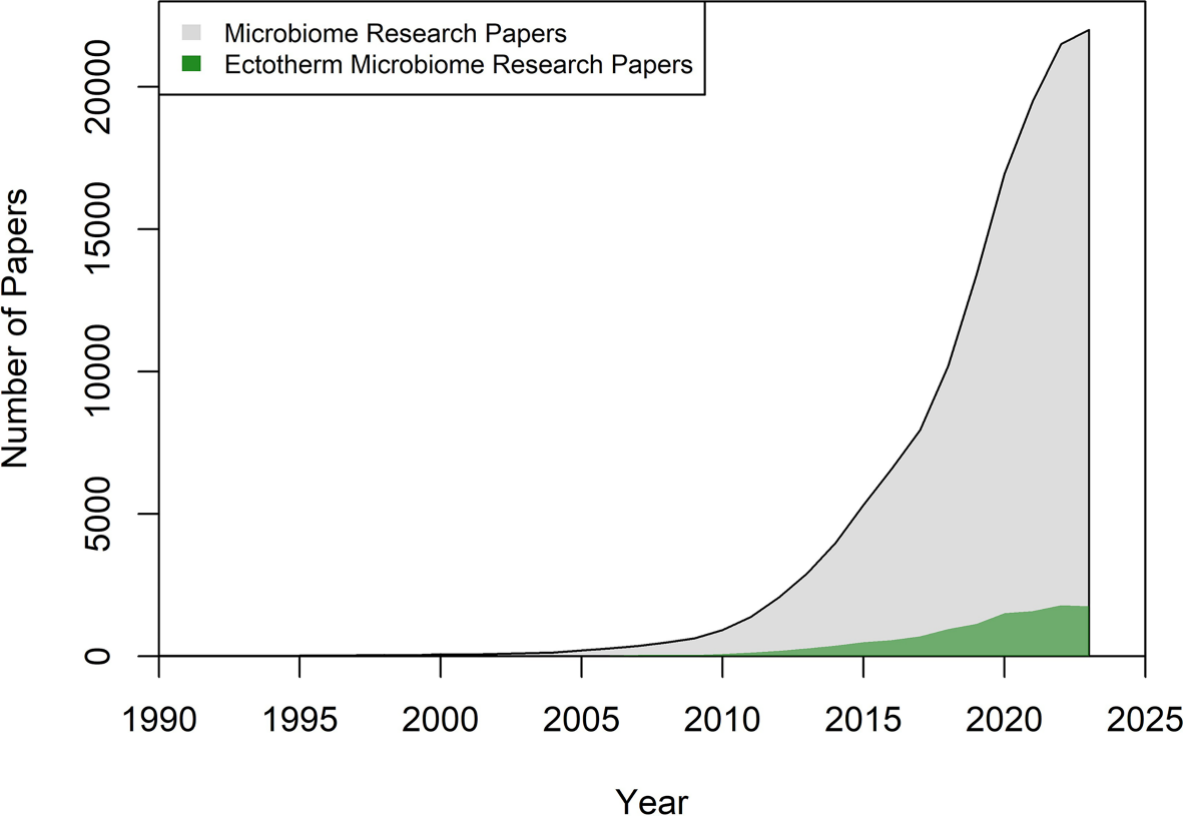
Primary research into the microbiome in the context of health has increased rapidly from the 2010’s but research on the microbiome of ectotherm species is slowing in proportion. Data on number of papers published sourced from PubMed. Papers looking into the microbiome identified by search query “(microbiome) OR (microbiota).” Microbiome papers investigating ectotherm species identified by search query “[(microbiome) OR (microbiota)] AND [(ectotherm) OR (amphibian) OR (reptile) OR (insect) OR (invertebrate) OR (fish)].” The quantities of review articles were deducted from each year of each query.

While research into animal microbiotas has grown exponentially until recently, 90% of this research has focused on homeotherms (Fig. 1) that make up <1% of animal species (Atkinson and Sibly 1997). Conversely, research into ectotherm microbiotas represents <10% of studies to date and seems to have reached its peak (Fig. 1). Moreover, our depth of knowledge on ectotherm microbiota compositions and functions is comparatively meagre, being informed by comparative studies of diseased or stressed vs healthy animal populations that yield limited mechanistic insight (Knutie et al. 2018, Tong et al. 2019, Yang et al. 2020b). Further investigations are thus needed to understand how environmental conditions affect ectotherm microbiomes to impact ectotherm fitness. This may lead to understanding microbiota composition and metabolic shifts associated with ectotherm health states (Basic et al. 2022), and to the knowledge needed to develop targeted pharmacomicrobiomics (Panebianco et al. 2018) and probiotic supplements (Liu et al. 2018) in support of ecologically and economically important species, or to enhancing conservation efforts for endangered species, such as by improving survival rates in captive breed and release programs (Yang et al. 2020a).

Whilst the value of further research into ectotherm microbiotas is acknowledged (Knutie et al. 2018, Tong et al. 2019, Yang et al. 2020b), its paucity may be explained by critical limitations. Endangered or economically relevant ectotherm species may not be studied in laboratory conditions, owing to fastidious husbandry or handling (e.g. natural environments hard to reproduce in a lab, low fecundity), expense, lack of fundamental knowledge on a species genome or ecology, lack of experimental tools (e.g. genetic modification), or ethical considerations (Mogil 2009, Pound and Ritskes-Hoitinga 2018, Holtze et al. 2021). There may also be insufficient economic or career incentives to develop them as model systems (Meigs et al. 2018).

Nevertheless, established animal models can help offset most of these costs, similarly to how they are utilized to study human disease mechanisms and develop therapeutics (Conn 2017). Here, we highlight the free-living nematode *Caenorhabditis elegans* as an emerging experimental model suitable for the study of ectotherms gut microbiotas and their interactions with their hosts. We review the history of *C. elegans* research in the context of host–gut microbiota studies, relevant tools and resources developed by the *C. elegans* community and propose experimental pipelines to accelerate research on nonmodel ectotherm gut microbiotas to reduce the knowledge gap with homeotherms on this topic.

## Main body

### Historic development of *C. elegans* as a microbiome model

**Figure 2:**
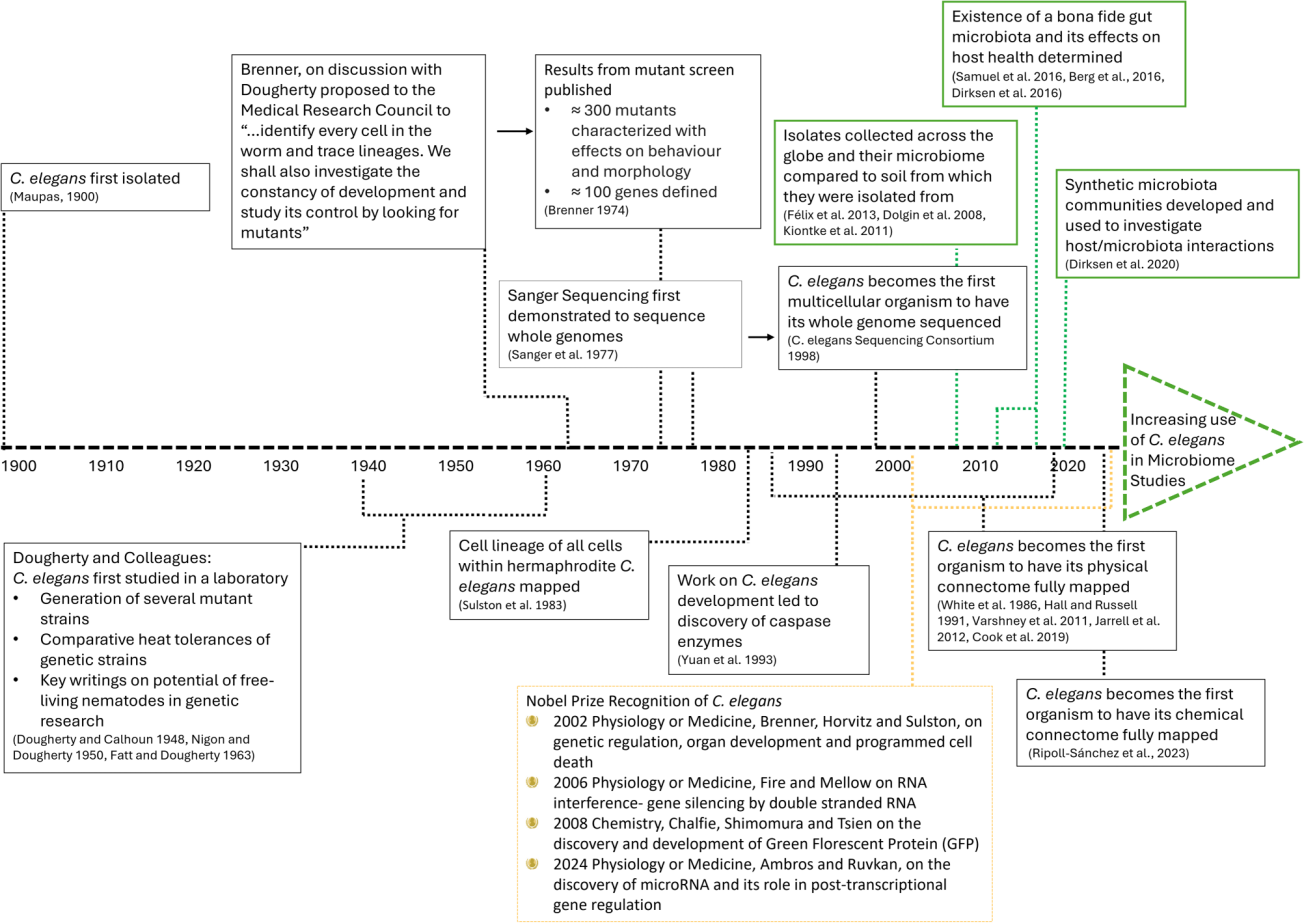
*Caenorhabditis elegans* was isolated long before its eventual utilization in the lab as a model for development, buat today it is a well-characterized, recognized, and widely used model organism in many fields, including the study of host–microbiome interactions. Black (…): general research with *C. elegans*. Green (…): microbiome centred research with *C. elegans*. Yellow (…): Nobel prize recognitions involving *C. elegans*.

*Caenorhabditis elegans* is a bacterivorous, soil litter-dwelling nematode, with over half a century as an experimental model organism (Fig. 2). From its first recorded isolation at the turn of the 20th century (Maupas 1900), *C. elegans* was brought to the laboratory in the 1940’s while aiming to identify a suitable nematode model species for genetic studies (Dougherty and Calhoun 1948). It was later defined as a key model organism to investigate developmental cell biology questions and understand how neuronal fate and connectivity drive animal behavior (White et al. 1997). Owing to its small genome (∼100 Mb), and demonstrated amenability to genetic interventions, *C. elegans* was notably selected for large collaborative endeavors, including the mapping of its physical and chemical connectomes (White et al. 1986, Hall and Russell 1991, Varshney et al. 2011, Jarrell et al. 2012, Cook et al. 2019, Ripoll-Sánchez et al. 2023), and the sequencing of its genome (C. elegans Sequencing Consortium 1998) ahead of that of humans (Nurk et al. 2022) and other animals (Adams et al. 2000, Chinwalla et al. 2002). Its ability to support fast-paced research across the biosciences led to major scientific discoveries, including four Nobel prizes to date, three in Physiology and Medicine (2002, “for discoveries concerning genetic regulation of organ development and programmed cell death”; 2006, “for the discovery of RNA interference—gene silencing by double-stranded RNA”; and 2024, “for the discovery of microRNA and its role in post-transcriptional gene regulation”) and one in Chemistry (2008, “for the discovery and development of the green fluorescent protein, GFP”) (The Nobel Prize 2002, 2006, 2008, 2024).

Meanwhile, interest into the native environment of *C. elegans* and its potential as a model for gut microbiota research began later, in 2008, with the Marie-Anne Felix lab collecting *C. elegans* wild isolates from across the globe to define its natural genetic and developmental diversity (Dolgin et al. 2008, Kiontke et al. 2011, Félix et al. 2013). This work laid the ground for the study of its native gut microbiotas, which culminated in 2016 with three seminal publications establishing that *C. elegans* adults host a *bona fide* gut microbiota (Berg et al. 2016, Dirksen et al. 2016, Samuel et al. 2016), and reporting varying effects of reconstituted model microbiomes on host health (Zhang et al. 2017a). *Caenorhabditis elegans* was notably found to bear a species-rich bacterial community dominated by the phylum *Proteobacteria*, recently renamed *Pseudomonadata* (Oren and Garrity 2021) including *Enterobacteriaceae* and members of the genera *Pseudomonas, Stenotrophomonas, Ochrobactrum, and Sphingomonas* (Dirksen et al. 2016), which was distinct from that of the microbial composition of the soils where it was isolated, and of that of its congeneric nematode species *C. remanei*. Reported gut microbiota compositions for *C. elegans* isolates that are genetically divergent differed, suggesting host genetic control on gut microbiota composition. Yet, meta-analyses of *C. elegans* natural microbiomes identified *C. elegans*-specific overlaps in gut microbiota phylogenetic composition, highlighting a dozen core fast-growing Gram-negative bacterial families (Zhang et al. 2017b), which led to the design of a consensus minimal model microbiota: CeMbio (Dirksen et al. 2020). Studying single gut microbiota isolate effects on *C. elegans* health (Samuel et al. 2016) identified both beneficial bacteria promoting stress-free growth, and detrimental bacteria that impaired growth, killed, or activated stress/immune reporters. Some *Proteobacteria*, including *Enterobacteriaceae* (*Enterobacter, Providencia*), *Gluconobacter*, and most *Lactococcus* strains were found to be beneficial to *C. elegans*. Conversely, detrimental genera belonged to the phylum *Bacteroidetes* such as *Chryseobacterium* and *Sphingobacterium* spp., and the class *Gammaproteobacteria* such as *Xanthomonas* and *Stenotrophomonas* spp.. These findings helped reach a consensus between the Felix, Samuel, Schulenburg, and Shapira teams when defining the core model gut microbiota for *C. elegans*: CeMbio (Dirksen et al. 2020). Amongst key considerations, the isolates chosen had to span across the 12 core bacterial types previously identified, be readily culturable, capable of colonizing the worm gut in monoxenic conditions and persist within the community, exhibit some metabolic complementarity, and not adversely affect the nematode’s life-traits. Other more or less complex model gut microbiotas are in use in different laboratories to study specific questions or to recapitulate more naturalistic conditions, such as the BIGbiome(Zhang et al. 2021).

With these defined gut microbiotas, *C. elegans* has become an effective metaorganism model in which to study causal relationships within a host–microbiome system. This has majorly included studies on the impact of gut commensal metabolism on host physiology relevant to human health (Watson et al. 2014, Feng et al. 2023, Zimmermann et al. 2024, Han et al. 2024) notably in the context of ageing (Miller et al. 2024), yielding results that echo findings in vertebrates, which includes the benefits of folate metabolism (Weinkove 2025) and short-chain fatty acids (Dalile et al. 2019, Walker et al. 2021, Bae et al. 2025), or detrimental effect of branched-chain fatty acids (Li et al. 2025) and more (Feng et al. 2023, Kim et al. 2024). It has also led to identifying mechanisms by which the native gut microbiota can protect worms from natural pathogens (Han et al. 2021), understanding how typically mutualistic bacteria can become pathogenic to *C. elegans*, and how pathogenic bacteria can lose virulence when another pathogen is present (Armitage et al. 2022). Other studies investigated how microbiota isolates perform mutually beneficial functions such as deactivating toxins and forming protective biofilms (Tang et al. 2023, Francisco and Grau 2025, Li et al. 2025c). Yet, investigations on how the gut microbiome can shield ectotherms, including *C. elegans*, against abiotic stressors and toxicants remain scarce. In particular, *C. elegans* high-throughput methodologies successfully applied to screening for probiotics and to studying host/microbiota/drug interactions with relevance to human health and ageing (Park et al. 2014, García-González and Walhout 2017, Rosa et al. 2023, Kang et al. 2024), could be exploited to inexpensively and rapidly explore the microbiotas of understudied ectotherms and design protectiveinterventions.

### Specific advantages of *C. elegans* to study host–gut bacterium interactions in nonmodel ectotherms

For the past 15–20 years, microbiome research has been largely driven by the promise of microbial therapies for chronic human diseases, especially metabolic, immunological, and mental health disorders, chiefly relying on mouse models to emulate human conditions (Franklin and Ericsson 2017, Knight et al. 2017). *Caenorhabditis elegans* is most commonly maintained in Petri dishes on Nematode Growth Medium agar seeded with a lawn of *Escherichia coli* strain OP50 at temperatures between 15°C and 25°C (Stiernagle 2006, Zhang et al. 2015). Presenting as either self-reproducing XX hermaphrodites or X0 males, *C. elegans* affords maintenance of isogenic populations, genetic crosses (Riddle et al. 1997), and can be cryopreserved (Brenner 1974, O’Connell 2022, Agrawal and Babu 2025), providing unrivalled control over host genetic make-up. Its short lifecycle and simple maintenance (Fay 2006) make them an inexpensive and versatile model to work. *Caenorhabditis elegans* supports straightforward protocols to manipulate both host and microbiota, to assign functions to microbial taxa, to conduct cost-effective experiments over short timescales, it enables complex and high-throughput experimental designs and it does not raise animal welfare issues susceptible to limit investigations (Douglas 2019). Against other powerful model metaorganisms such as *Drosophila melanogaster* (fruit fly), *Danio rerio* (zebrafish), and *Mus musculus* (mouse), *C. elegans* uniquely offers unmatched levels of experimental control on both host and microbiota culture and modification, while affording large-scale screening of microbiota isolates (Ali et al. 2022) and testing of host–microbe interactions (Table 1).

**Table 1:** Key characteristics of most developed experimental animal models for gut microbiota studies.

Feature	*C. elegans*	*D. melanogaster*	*D. rerio*	*Mus* sp.	References
Host internal temperature	Ectotherm, 12°C–26°C (1)	Ectotherm, 11°C–32°C (2)	Ectotherm, 6°C–38°C (3)	Homeotherm, 36.2°C–37.5°C (4)	(1) Zevian and Yanowitz (2014)(2) Rivera-Rincón et al. (2024)(3) Beitinger et al. (2000)(4) Russel et al. (2021)
Mode of feeding	Bacterivorous	Saprophagous	Omnivorous	Opportunistic omnivorous	
Laboratory diet(s)	*E. coli* OP50 as standard, HT115, NA22, or HB101 also common; defined diets available (1, 2)	Live yeast + sugar/cornmeal + agar typically in varying proportions; defined diets available (3)	Commercial flakes or micropellets; no standardized diet (4)	Chow (seed and plant-based typically) or defined diet	(1) Zecic et al. (2019)(2) Flavel et al. (2018)(3) Lesperance and Broderick (2020)(4) Hillman et al. (2024)
Native gut microbiota complexity	Very low; largely aerobic bacteria (*Enterobacteriaceae, Pseudomonadaceae, Xanthomonadaceae*, and *Sphingobacteriaceae*), no evidence of fungi, archaea, or viruses. Other nematodes have more complex microbiotas (1)	Low, dominated by bacteria (*Acetobacteraceae*, Lactobacilli, *Leuconostocaceae, Enterococaceae*, and *Enterobacteriaceae*), also includes fungi (yeasts mainly) and viruses (2)	Low, mostly bacteria (*Pseudomonadota, Fusobacteriota* (previously *Fusobacteria), Bacillota* (prev. *Firmicutes), Bacteroidota* (prev. *Bacteroidetes), Actinomycetota* (prev. *Actinobacteria*), and *Verrucomicrobia*), but also fungi, archaea and viruses (3, 4)	High, bacteria (*Bacillota, Bacteroidota, Spirochaetota, Pseudomonadota, Deferribacterota, Actinomycetota*, Cyanobacteria, *Saccharibacteria, Mycoplasmatota, Verrucomicrobiota, Elusimicrobiota, Fusobacteriota*, fungi, archaea, (including methanogens), and viruses (5, 6)	(1) Trejo-Meléndez and Contreras-Garduño (2025)(2) Ludington et al. (2025)(3) Roeselers et al. (2011)(4) Thormar et al. (2024)(5) Yang and Chun (2021)(6) Chu and Zhang (2022)
Native gut microbiota culturability	Excellent (>99%) (1)	Good (>80%) (2)	Fair (∼50%) (3)	Poor (∼23%–65%) (4, 5)	(1) Dirksen et al. (2020)(2) Ludington et al. (2025)(3) Cantas et al. (2012)(4) Lagkouvardos et al. (2017)(5) Beresford-Jones et al. (2022)
Gnotobiotic animal generation	Easy and inexpensive (1)	Relatively easy and inexpensive (2, 3)	Relatively easy in larvae, harder for adults (4)	Challenging and costly, requiring embryo transplant in germ free mothers or caesarian birth, and raising in germ-free grade facilities (5, 6)	(1) Backes et al. (2021)(2) Koyle et al. (2016)(3) Wu et al. (2023)(4) Xia et al. (2022)(5) Arvidsson et al. (2012)(6) Zucoloto et al. (2021)
Availability of model gut microbiotas	CeMBio (1); BIGbiome (2)	No consensus model (3)	No consensus model (4)	ASF (5); HMA (6)	(1) Dirksen et al. (2016)(2) Zhang et al. (2021)(3) Ludington and Ja (2020)(4) Zhong et al. (2022)(5) Lyte et al. (2019)(6) Arietta et al. (2016)
Lifecyle (age to sexual maturity)	2.5 days at 25°C, 5 days at 15°C	10 days at 25°C	10–12 weeks at 28.5°C	6 weeks (females); 8 weeks (males)	
Typical Lifespan (median) in the lab	17–21 days at 20°C	60–70 days at 25°C	2–4 years	18–24 months	
Experimental scalability	Very high	High	High	Low	
Cryopreservation	Larval stages L1, L2, L2d, and dauer can be cryogenically frozen (1)	Embryo can be cryogenically frozen (2)	Sperm and embryos can be cryogenically frozen	Sperm and embryos can be cryogenically frozen	(1) Stiernagle (2006)(2) Zhan et al. (2021)
Genetic resources	Genome-wide mutant, transgenics and RNAi libraries (>26 300–Caenorhabditis Genetics Center); >13 500–National BioResource Project; > 20 000 RNAi—Horizon Discovery)(1, 2, 3)	Extensive mutant and transgenics libraries (>175 lines—Drosophila Genomics Resource Center; >15 500–NBRP) (4, 5)	Extensive mutant, shRNA and morpholino libraries (>30 000–EZRC; >44 000–ZIRC; morpholinos—Gene Tools, LLC (6, 7, 8)	Extensive mutant and shRNA libraries (>9000–EMMA; >22 000–MMRRC; shRNA library—The RNAi Consortium; (9, 10, 11)	(1) cgc.umn.edu/ (2) shigen.nig.ac.jp/c.elegans/ (3) horizondiscovery.com/en/non-mammalian-research-tools/products/c-elegans-rnai-collection(4) dgrc.bio.indiana.edu/Home(5) fruitfly.jp/flystock/about_db_e. (6) ezrc.kit.edu/ (7) zebrafish.org/ (8) gene-tools.com/ (9) infrafrontier.eu/emma/ (10) mmrrc.org/ (11) Coussens et al. (2011)
Maintenance cost	Low	Low	Medium	High	

#### *Caenorhabditis elegans* affords large, gnotobiotic, age-synchronized populations for omics studies

Studying host–microbe interactions requires control over the biotic environment of the animal model, which is then called gnotobiotic. Typically, it requires generating germ-free animals before inoculating them with defined microbial cultures. In *C. elegans*, bleach treatment of gravid hermaphrodites releases sterilized eggs that can be used to generate gnotobiotic models that are either axenic/germ-free (Avery 1993), monoxenic (Stiernagle 2006), or polyxenic (Dirksen et al. 2020). With *C. elegans* hermaphrodites laying 4–10 eggs per hour (Schafer 2005), yielding 100–120 eggs/day at peak reproduction, and a brood size of 200–300 for unmated hermaphrodites (Hodgkin et al. 1979, Kwah and Jaramillo-Lambert 2023) and ∼1000 for mated hermaphrodites (Hodgkin and Barnes 1991), large age-synchronized populations are easily obtained for host–microbiota “omics” studies (Tamez González et al. 2024).

**Figure 3:**
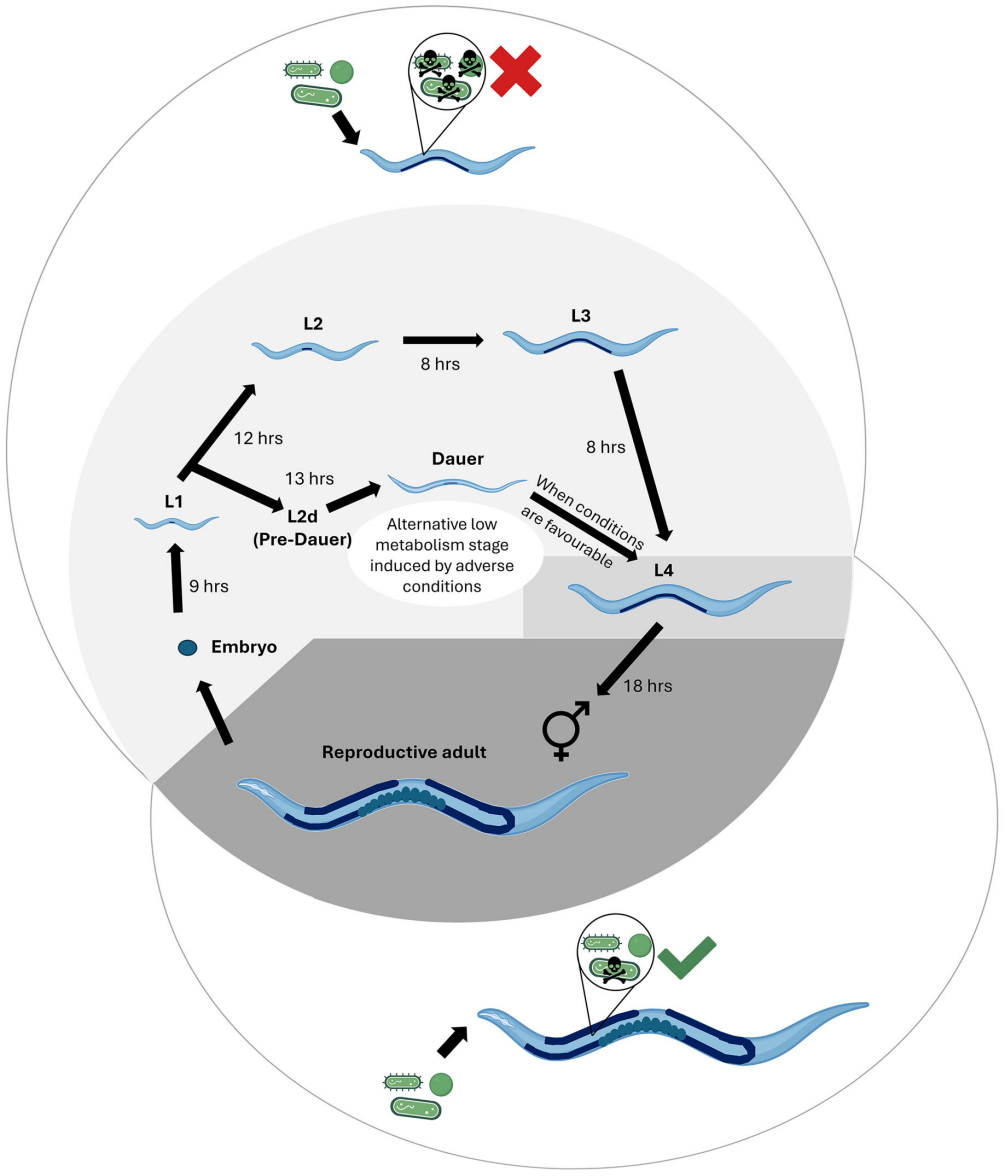
The bacterivorous *C. elegans* short life cycle comprising four moult stages only affords de novo gut microbiota establishment in adulthood. Developmental timepoints refer to development at 20°C. The relative lengths of the developmental stages depicted are proportionally accurate. Illustration created using BioRender and Microsoft Powerpoint.

As a bacterivorous species, *C. elegans* grinds and kills ingested bacteria using pharyngeal teeth (Albertson and Thompson 1976). Consequently, bacteria appear unable to colonize the worm gut before the fourth larval stage or adulthood depending on the strain, possibly exploiting enlarged gaps between pharyngeal teeth to reach the gut alive (Portal-Celhay et al. 2012, Zhang et al. 2021, Kissoyan et al. 2022; Fig. 3). This suggests that a *bona fide C. elegans* gut microbiota only establishes naturally in adulthood (Dirksen et al. 2020, Zhang et al. 2021). It also provides a tighter control over the composition of the worm gut microbiota for experimental studies. However, the dual role of gut bacteria as both main nutrition and symbiote can confuse interpretations of worm–gut bacterium interactions. Therefore, the *C. elegans* community has developed approaches to disentangle the dietary versus metabolic or signaling contributions of microbiotas to host health. These include feeding worms defined media, which helped determine the nutritional requirements and metabolic flexibility of *C. elegans* (Szewczyk et al. 2003, Castelein et al. 2008, Flavel et al. 2018, Zhu et al. 2025), or inactivated bacteria that are freeze-dried, irradiated, heat-killed or paraformaldehyde-treated (Lenaerts et al. 2008, Beydoun et al. 2021, Gao et al. 2024). These approaches and the knowledge they generated notably affords feeding a complete diet to naïve worms before exposing them to a gut bacterium of interest (Chelliah et al. 2018).

#### *Caenorhabditis elegans* enables high-throughput quantitative host phenotyping of gut microbial influences

Another desirable attribute for a reductive model that empowers microbiota–gut–brain axis investigations in the context of ectotherm resilience, is amenability to rapid and quantitative phenotyping. *Caenorhabditis elegans* small size, short life cycle and lifespan (Table 1) permit faster and high-throughput life-trait, metabolic and behavioral analyses (Cornaglia et al. 2017) in microplates, microfluidics, and other formats, including automated measurements of feeding (Bonnard et al. 2022), respiration (Koopman et al. 2016, Haroon and Vermulst 2019), developmental rate (Olmedo et al. 2015), reproduction (Peng et al. 2025), ageing, lifespan, and healthspan (Stroustrup et al. 2013, Churgin et al. 2017, Pittman et al. 2017, Felker et al. 2020, Rahman et al. 2020, Barlow et al. 2022, Gardea et al. 2022, Zavagno et al. 2024), and stress and infection resistance (Benedetto et al. 2019). Thanks to the small footprint of *C. elegans* assays, aseptic robotized labs can be built to automate multiassay phenotyping for longitudinal studies on thousands of compounds in parallel (Fahs et al. 2025). Such automated methodologies have been successfully applied to evaluate tripartite interactions between host, microbiota, and drugs across many conditions in parallel (García-González et al. 2017, Scott et al. 2017a). Bacterial intake and gut clearance can be measured by time-lapse microscopy recording pharyngeal pumping and defecation rates, which have also been successfully automated (Wibisono and Sun 2022). Similarly, locomotion direction, speed, and other behavioral events in response to bacteria or other stimuli can also be tracked and related to neuronal and muscular functions (Laranjeiro et al. 2019, Lee et al. 2024, Petratou et al. 2024, Hernandez-Lima et al. 2025).

#### A genetically tractable model for the mechanistic dissection of host–gut microbiota interactions

*Caenorhabditis elegans* genetic tractability, amenability to genetic manipulations, and stereotypical anatomy uniquely affords establishing causal relationships in host-gut microbial interactions (Dirksen et al. 2016, 2020, Park et al. 2020, Viri et al. 2020, Haçariz et al. 2021, Taylor and Vega 2021, Shi et al. 2022, Espejo et al. 2024).

With the capacity to either carry out genetic crosses or use self-fertilization, *C. elegans* provides increased genetic flexibility compared to other models. Outcrossing provides the ability to generate traditional multimutant and multitransgenic animals, while self-fertilization minimizes genetic variation between progenies that are then typically isogenic for all loci. This notably permits the study of nervous system mutants with serious locomotion defects that in other animals would prevent reproduction (Choi et al. 2015, Gottschling et al. 2017). This genetic flexibility has also allowed the rapid generation of isogenic outbred lines for rapid genetic mapping of genetic variations of interest (Doroszuk et al. 2009, Jaramillo-Lambert et al. 2015). Critically, *C. elegans* genetic stocks can be preserved for decades so that laboratories worldwide can keep performing experiments in the same genetic background, including the original wild-type strain Bristol N2, frozen in 1973 (Brenner 1974, Sterken et al. 2015). Indeed, 20%–90% of L1, L2, L2d, and dauer stage animals can survive one freeze–thaw cycle, depending on factors such as freezing solution composition and feed state (Stiernagle 2006, Agrawal and Babu 2025). This means that since the 1970s, a vast collection of genetically diverse wild, mutant, interbred, and transgenic strains has been assembled and made available to the scientific community worldwide, available from the Caenorhabditis Genetic Center at the University of Minnesota (https://cgc.umn.edu/), which grew from 3000 strains in 2006 (Stiernagle 2006) to over >26 000 currently, and the Japanese National BioResource Project laboratory currently with >13 500 strains (https://shigen.nig.ac.jp/c.elegans/top.xhtml).

Another useful idiosyncrasy of *C. elegans* is its sensitivity to RNA interference (The Nobel Prize 2006), which can be conveniently delivered by dietary *E. coli* producing 500–1000 bp double-strand RNA (Kamath and Ahringer 2003, Conte et al. 2015). This has led to the generation of shareable genome-wide RNAi libraries (Kamath et al. 2003, Rual et al. 2004, C. elegans RNAi Collection 2026.). Like other animal models, *C. elegans* can be genetically manipulated to express foreign genetic sequences using a variety of transformation techniques leading to transgene overexpression (Fire and Waterston 1989, Mello et al. 1991), mosaic expression (Yochem et al. 1998), or single-copy insertions and modifications by engineered transposable elements (Frøkjær-Jensen et al. 2008, Robert et al. 2009), or CRISPR-Cas9 (Waaijers and Boxem 2014), depending on experimental requirements. Using *C. elegans* mutants or RNAi together with microbiota libraries, host phenotypic screens can be performed to genetically dissect host–gut microbiota interactions (Ali et al. 2022, Zárate-Potes et al. 2022). For example, mutation of the *C. elegans* catalase (*ctl-2*) was found to abolish *Bifidobacterium adolescentis* benefits to their host (Chen et al. 2021). Confirmed by mouse and fruit fly studies, it revealed a conserved functional interaction between *B. adolescentis* and host regulation of CAT activity and metabolism.

Synergizing with its amenability to genetic engineering, *C. elegans* exhibits a largely invariant cell lineage and stereotypical anatomy, where each of the hermaphrodite 959 somatic cells occupies the same position, adopts the same fate and carries out the same role in every individual (Alberts et al. 2002). With its fixed cell number, *C. elegans* also lacks the ability to replace somatic cells, which enables cell ablation studies by physical (Chapter 10 Laser Killing of Cells in *Caenorhabditis elegans*^1995^) or genetic (Qi et al. 2012, Williams et al. 2013) means to determine the exact contribution of individual cells and their descendants to the process under study (Meneely et al. 2019). Such approaches are more targeted than knocking-out transcription factors that may erase entire cell lineages or have “off target effects” (Kimble 1981). For instance, single cell ablation and tissue-specific knock-down of immunity genes have allowed mapping of worm neurones detecting bacterial quorum-sensing molecules and microbe-specific host responses (Werner et al. 2014, Zheng D et al. 2020).

#### *Caenorhabditis elegans* transparency affords live monitoring of gut microbiota dynamics in vivo

Host–gut microbiota interactions are highly dynamic with microbiota composition and associated host physiological states varying greatly between individuals and across the lifespan (Spor et al. 2011, Chang and Kao 2019). Most experimental animal models afford sampling of gut microbiota over time from faeces, or catheters, with associated host health phenotyping (Inamine et al. 2018, Gaulke et al. 2019, Santos et al. 2021, Thormar et al. 2024, Sommer et al. 2025). However, continuous monitoring and direct visualization of host–gut microbe interactions is currently out of reach, limiting our ability to resolve the dynamics of host–gut microbe interactions. Thanks to *C. elegans* transparency, fluorescently tagged gut bacteria can be visualized by time-lapse fluorescence microscopy (Tan et al. 1999, DebRoy et al. 2012, Kissoyan et al. 2022). In parallel, *C. elegans* offers many genetically encoded reporters to track its physiological responses to microbes (Estes et al. 2010, Julien-Gau et al. 2014, Gravato-Nobre et al. 2016, Bar-Ziv et al. 2020). *Caenorhabditis elegans* small size also means that it can be handled on slides, plates or in-chip, and is compatible with microfluidics-assisted high-throughput imaging. With a thickness of 60–70 um, adult *C. elegans* and their tissues are particularly well-suited for live high-resolution volumetric imaging by a variety of light imaging modalities including confocal scanning-laser (White et al. 1987), spinning-disk (Labbé et al. 2003), light-sheet (Van Krugten et al. 2021), and lattice-light sheet (Chen et al. 2014) fluorescence microscopy (Shah et al. 2022). Together, these attributes uniquely afford tracking of both microbial colonization/composition changes and host immune, metabolic, and stress responses with excellent spatiotemporal resolution *in vivo*.

### Key considerations for using *C. elegans* as a model system to study host–microbiota interactions

Whilst *C. elegans* provides specific advantages over other models for gut microbiota studies, it is important to highlight existing limitations (Table 2). As gut microbiotas are species-specific, *C. elegans* has a predilection for specific microbes, although they overlap with other organisms at phylum level (Table 1). Its native gut microbiota is also simpler and has only been found to contain aerophilic bacteria, given that the worm is tens of microns thick and oxygen diffuses readily to its gut (Berg et al. 2016, Dirksen et al. 2016, Samuel et al. 2016). Thus, its lack of coevolution with other types of gut microbes, including yeasts and other fungi, may limit its applicability when investigating other major compartments from larger animal gut microbiotas. Even when restricting the application of the worm model to studying host interactions with gut bacteria, further considerations are needed.

**Table 2:** Model-specific advantages and disadvantages of *C. elegans* as a model animal in host–gut microbiota studies.

Category	Feature	Advantages	Limitations for microbiota studies	References
Laboratory handling	Bacterivorous diet	Bacteria ground and killed by pharyngeal teeth (1), limiting gut colonization to latest larval stage or adulthood (2, 3, 4, 5, 6) facilitates gnotobiotic setups	Late gut colonization (2, 3, 4, 5, 6) prevents studies of host developmental modulation by gut bacterial microbiota Dual role of bacteria as gut symbiont and nutrition can confound interpretations of worm–gut bacterium interactions	(1) Albertson and Thompson (1976)(2) Portal-Celhay et al. (2012)(3) Zhang et al. (2021)(4) Kissoyan et al. (2022)(5) Dirksen et al. (2020)(6) Zhang et al. (2021)
Laboratory handling	Cryopreservation (1, 2)	Extensive strain libraries (genetically diverse wild, mutant, interbred, and transgenic) (3, 4) Preservation of genetic/epigenetic backgrounds circumvents genetic drift issues (5, 6)	Freeze–thaw is a stress that impacts worm physiology (7, 8). Stressed worms may take multiple generations to recover transcriptomically (9)	(1) Stiernagle (2006)(2) Agrawal and Babu (2025)(3) https://cgc.umn.edu/(4) https://shigen.nig.ac.jp/c.elegans/top.xhtml(5) Zhan et al. (2021)(6) O’Connell (2022)(7) Stastna et al. (2020)(8) Hu et al.(2015)(9) Rechavi et al. (2014)
Laboratory handling	Working aseptically	Bleach treatment of gravid hermaphrodites releases sterile eggs to establish gnotobiotic models (1, 2, 3) Defined diets and inactivated bacteria afford maintenance in aseptic conditions (4, 5, 6, 7, 8, 9, 10)	Bleach exposure stresses worms born from bleached eggs (11) but filtration-based protocols can be used (12) Defined diet and inactivated bacteria may not fully recapitulate natural host physiology (13, 14)	(1) Avery (1993)(2) Stiernagle (2006)(3) Dirksen et al. (2020)(4) Szewczyk et al. (2003)(5) Castelein et al. (2008)(6) Flavel et al. (2018)(7) Zhu et al. (2025)(8) Lenaerts et al. (2008)(9) Beydoun et al. (2021)(10) Gao et al. (2024)(11) Verdú et al. (2022)(12) Jhaveri et al. (2025)(13) Schulenburg and Félix (2017)(14) Beydoun et al. (2023)
Laboratory handling	Temperature	Extensive range of protocols and knowledge on the effects of temperature on worm physiology throughput the lifespan (1, 2, 3, 4, 5, 6)	Transiently survives 4°C–33°C, reproduces between 12°C and 26°C (7), traditionally maintained at 16°C–20°C: cannot be used to fully replicate host–microbe interactions outside these temperature ranges	(1) Goodman and Sengupta (2019)(2) Kim et al. (2020)(3) Mori et al. (2025)(4) Ali et al. (2022)(5) Walker et al. (2001)(6) Morley and Morimoto (2004)(7) Zevian and Yanowitz (2014)
Genetics	Genome	Self-reproducing XX hermaphrodites affords generation and maintenance of isogenic strains (1) X0 males allow for easy genetic crossing	No true clones, individual variability is still significant (2)	(1) Brenner (1974)(2) Barrière and Félix (2005)
Genetics	Transgenesis	Possibility to express foreign genetic sequences from extrachromosomal arrays for transgene overexpression (1, 2, 3) or mosaic expression (4), via single-copy insertions from transposable elements (5, 6) or CRISPR-Cas9 (7) Multiple for methods for transgene delivery (e.g. gold-particle bombardment, injection) (8) The *C. elegans* genome can accept large amounts of foreign material [e.g. 41 transgenes in the NeuroPAL strain, (9)]	Extrachromosomal arrays may suffer from germline silencing (10) Transgenic worms often superficially wild type may be physiologically different (8) and may interact with bacteria differently	(1) Mello et al. (1991)(2) Fire and Waterston (1989)(3) Rich et al. (2025)(4) Yochem et al. (1998)(5) Frøkjær-Jensen et al. (2008)(6) Robert et al. (2009)(7) Waaijers and Boxem (2014)(8) Nance and Frøkjær-Jensen (2019)(9) Yemini et al. (2021)(10) Aljohani et al. (2020)
Genetics	RNA interference	Availability of shareable genome-wide RNAi libraries (1, 2, 3) RNAi treatment easily administered by soaking (4), injection (5) or by feeding dsRNA-expressing *E. coli* (6, 7) Tissue-specific RNAi is possible via different methods (8)	*E. coli-*produced RNAi must be delivered either by live microbes prior to microbiota being introduced (9) or *E. coli* RNAi bacteria need to be inactivated when fed alongside gut microbiotas	(1) Kamath et al. (2003)(2) Rual et al. (2004)(3) C. elegans RNAi Collection (2026)(4) Maeda et al. (2001)(5) Fire and Waterston (1989)(6) Kamath and Ahringer (2003)(7) Conte et al. (2015)(8) Watts et al. (2020)(9) Zarate-Potes et al. 2022)
Anatomy	Organ system	Simple well defined transparent organs easy to monitor over time (1, 2, 3, 4)	Absence of cardiovascular and respiratory systems owing to small size (5)	(1) Garigan et al. (2002)(2) Kormish et al. (2010)(3) Ezcurra et al. (2018)(4) Buechner et al. (2020)(5) Corsi et al. (2018)
Anatomy	Gut physiology	Single-cell layer epithelial tube with microvilli (1) likely forms a single microbiota niche (2, 3, 4) simplifying host gut–microbe interactions studies pH oscillations between 3.6 and 6 (5) recapitulate pH ranges experienced in other gut microbiotas	Lack of anatomical gut compartmentalization (1) Narrower pH range compared to other species limits applicability of *C. elegans* for acidophilic (6) or alkaliphilic (7, 8) bacteria species *C. elegans* small size does not afford anaerobic gut conditions (9), limiting the study of exclusive anaerobes or facultative anaerobes in anaerobic *in vivo* conditions *C. elegans* narrow gut lumen (1) limits establishment of sharp metabolite, nutrient, gas, and solute gradients observed in other hosts	(1) McGhee (2007)(2) Berg et al. (2016)(3) Dirksen et al. (2016)(4) Ortiz et al. (2021)(5) Chauhan et al. (2013)(6) Bik et al. (2006)(7) Thongaram et al. (2003)(8) Liang et al. (2018)(9) Bargmann (2006)
Anatomy	Nervous system	Simple nervous system <350 cells with main neuronal pathway conserved affords rapid investigation of the microbiota gut-brain axis (1)	Glia differs in morphology, function, and lineage from that of vertebrates making translation of *C. elegans* findings less straightforward (2, 3)	(1) Ortiz de Ora and Bess (2021)(2) Stout Jr. et al. (2014)(3) Oikonomou and Shaham (2011)
Anatomy	Cell lineage	Invariant cell lineage and cell ablation techniques (1,2, 3, 4) afford cell-resolved dissection of host-gut microbe interactions (5) Absence of somatic stem cells (6) means that host cell renewal does not confound host pathology analyses in host-gut microbe interactions	*C. elegans* lacks key vertebrate cell types involved in host–gut microbiota including gut stem cells, circulatory and secondary immune cells (7) Host–gut microbe signaling that modulate host cell division rates (8, 9) cannot be recapitulated in *C. elegans*	(1) Chapter 10 Laser Killing of Cells in Caenorhabditis elegans (1995)(2) Qi et al. (2012)(3) Williams et al. (2013)(4) Kimble (1981)(5) Otarigho et al. (2024)(6) Herman (2006)(7) McGhee (2007)(8) Shin et al. (2022)(9) Cheesman et al. (2011)
Natural interactions with bacteria	Bacterial pathogens	Core innate immune pathways are conserved in *C. elegans* despite notable differences (1) *C. elegans* shares phylogenetically conserved pathogens and is sensitive to other animals’ pathogens (2, 3) *C. elegans* detects bacterial quorum-sensing cues (4)	Key adaptive immunity and inflammation pathways are absent in *C. elegans* (5) but *C. elegans* innate immunity is adaptive (6)	(1) Martineau et al. (2021)(2) Couillault and Ewbank (2002)(3) Darby (2005)(4) Werner et al. (2014)(5) Pukkila-Worley and Ausubel (2012)(6) Tran and Luallen (2024)
Natural interactions with bacteria	Bacterial commensals	Large collection of natural commensals affording studying combinatorial effects of gut bacterial isolates [CeMBio (1), BIGBiome (2)] Many worm bacterial commensal have sequenced and annotated genomes Key gut commensals are shared from *C. elegans* to vertebrates [e.g. *Acinetobacter guillouiae, Enterobacter hormaechei, Stenotrophomonas indicatrix*, and *Lelliottia amnigena* (4)]	Some key vertebrate gut commensals are not *C. elegans* natural commensals (e.g. *E. coli, E. faecalis, E. faecium, P. aeruginosa*… (4, 5) and worm interactions with these bacteria may not reflect the coevolved processes in natural hosts Some worm bacterial commensal genomes have not been sequenced yet, limiting our ability to exploit dual-omics methodologies	(1) Foltz and Herman (2025)(2) Zhang et al. (2021)(3) Zimmermann et al. (2020)(4) Dirksen et al. (2016)(5) Ley et al. (2008a)
Quantitative phenotyping	Bioimaging	Transparent body allows for live imaging of internal organs and microbes (1, 2) Small size affords versatile whole animal high-resolution imaging of multiple animals (2, 3) Low thickness (<75um) makes them suitable for volumetric imaging by most light imaging modalities and artefact-free high pressure freezing for ultrastructural studies (2, 4, 5)	Microscopes are required to manipulate and observe *C. elegans*, which incurs significant capital costs and access to microscopy facilities	(1) Breimann et al. (2019)(2) Rezaeianaran and Gijs (2023)(3) Kumar et al. (2019)(4) Manning and Richmond (2015)(5) Rostaing et al. (2004)
Quantitative phenotyping	Life-trait analyses	Fast stereotypical development affords rapid study of dietary, genetic and environmental impact on development (1, 2) Short lifespan affords rapid study of the role of host–gut–microbiota interactions in ageing (3) Fast life cycle accelerates study of transgenerational effects of diet, stress, and microbiota influences (4, 5, 6)	The worm fast life cycle and short lifespan may not afford studying interactions and processes that typically establish over weeks or month in other hosts	(1) Olmedo et al. (2015)(2) Xiao et al. (2015)(3) Miller et al. (2024)(4) Minkina and Hunter (2018)(5) Ivimey-Cook et al. (2021)(6) Sengupta et al. (2023)
Quantitative phenotyping	Omics approaches	Dual-RNA sequencing has been successfully achieved to study worm–gut microbe interactions (1, 2) *C. elegans* is amenable to population (3), single-animal (4, 5), and single-tissue (6) proteomics *C. elegans* affords untargeted and targeted metabolomics approaches relevant to studying host–bacterium interactions (7, 8, 9)	Dual-omics approaches require sequenced and annotated genomes for the relevant bacterial species, which may not be available Tissue-specific metabolomics are challenging to achieve in *C. elegans* and relies on single-cell omics predictions (10)	(1) Zhang et al. (2020)(2) Legüe et al. (2021)(3) Schrimpf and Hengartner (2010)(4) Bensaddek et al. (2016)(5) Zhu et al. (2024)(6) Tan et al. (2024)(7) Molenaars et al. (2021)(8) Helf et al. (2022)(9) Feng et al. (2023)(10) Yilmaz et al. (2020)
Quantitative phenotyping	Automated assays	*C. elegans* small size, short life cycle, and lifespan permit fast, high-throughput, often automated life-trait, metabolic and behavioral analyses e.g. feeding (1), respiration (2, 3), developmental rate (4), reproduction (5), ageing, lifespan, and healthspan (6, 7, 8, 9, 10, 11, 12, 13) and stress and infection resistance (14)	High-throughput automation in multiwell arrays and microfluidics may require liquid handling, which impacts worm metabolism (15, 16)	(1) Bonnard et al. (2022)(2) Koopman et al. (2016)(3) Haroon and Vermulst (2019)(4) Olmedo et al. (2015)(5) Peng et al. (2025)(6) Stroustrup et al. (2013),(7) Churgin et al. (2017)(8) Pittman et al. (2017)(9) Felker et al. (2020)(10) Rahman et al. (2020)(11) Barlow et al. (2022)(12) Gardea et al. (2022)(13) Zavagno et al. (2024)(14) Benedetto et al. (2019)(15) Çelen et al. (2018)(16) Lev et al. (2019)

#### Limits in *C. elegans* temperature tolerance range restricts gut microbiota studies

First, bacteria are highly sensitive to environmental conditions such as pH (Krulwich et al. 2011), osmolarity (Csonka 1989, Poolman and Glaasker 1998), and temperature (Moon et al. 2023). Hence, host temperature majorly determines gut microbial isolate composition across animal species (Sohail et al. 2015, Fontaine et al. 2018, Kokou et al. 2018, Thapa et al. 2019). Compared to endotherms that typically maintain body temperatures between 36°C and 38°C, or extreme cases in other ectotherms that experience much higher (>46.4°C basking lizards; Brattstrom 1965) or lower internal temperatures (<4°C overwintering subarctic wood frog; Costanzo et al. 2013), *C. elegans* may transiently tolerates 4°C–33°C but can only reproduce between 12°C and 26°C (Zevian and Yanowitz 2014) and is traditionally maintained at 16°C–20°C. Hence, *C. elegans* studies of ectopic gut bacterial species are restricted to that temperature range, where some isolates may display altered metabolisms that do not reflect their function(s) in the original host. In turn, it may curtail our ability to extrapolate findings from experiments involving worms with other ectotherm microbes, to their original host. To mitigate against this, when using *C. elegans* to model endotherm host–microbe interactions, experiments are carried out at the upper end of the worm’s tolerance range (25°C), which has proved sufficient to help elucidate conserved pathogenicity mechanisms of human commensals (Garsin et al. 2001a, Sifri et al. 2002, Irazoqui et al. 2010). In the context of most ectotherm gut microbiota studies, the worm’s natural temperature tolerance range is well-suited to studying the gut microbiotas of many temperate species.

#### Considerations on gut bacterium persistence in the worm gut

Persistence of introduced bacteria (e.g. probiotics) in a host gut is a frequent challenge across microbiota studies. Prebiotics may be used to support the newly introduced microbes, promoting their proliferation or adhesion to the mucosal lining of the intestine (You et al. 2022). However, in both healthy and diseased individuals, probiotic persistence rarely exceeds 2 weeks (Pavan et al. 2003, Horz et al. 2007, Tremblay et al. 2023) and current evidence suggests that both short- and long-term probiotic supplementation may lead to adverse side effects in mammals (Pace et al. 2020, Hradicka et al. 2023). Both human probiotic trials and *C. elegans* gut microbiota studies have also demonstrated that probiotic effects are dose-dependent and thus require probiotic persistence (Szulińska et al. 2018, Kumaree et al. 2023). With *C. elegans* being easily refed defined bacterial compositions as needed, persistence is not a concern to study worm-ectopic gut bacterium interactions, but findings may not easily be replicable in the target organism.

#### *Caenorhabditis elegans* simpler gut anatomy and physiology do not fully recapitulate other ectotherms’

Gut anatomies and physiologies vary greatly across animal species, as does gut compartmentalization associated with distinct niches that locally dictate specific gut microbial compositions (Donaldson et al. 2016, Mallott and Amato 2021, McCallum and Tropini 2024). In mammals, changes in the gut lumen environment such as digestion rate, transit time, physical pressures, pH, osmolarity, enzymatic composition, and metabolites, impact the metabolic fate of ingested nutrients and induce different selective pressures on microbiota present in the different segments of the intestine (Feng et al. 2019, Su et al. 2024, Zhou et al. 2024). By contrast, *C. elegans* intestine is a single tube believed to provide a single microbiota niche (Berg et al. 2016, Dirksen et al. 2016, Ortiz et al. 2021), much simpler than the gut of vertebrate ectotherms and arthropods, despite evidence of functional regionalization based on differential spatial expression of key transporters (Schroeder and McGhee 1998, McGhee 2007, Au et al. 2009). Additionally, many vertebrates that consume food followed by a period of fasting have a lower gastric pH than those that eat continuously, leading to differences in microbiota composition (Karasov and Douglas 2013). For example, snakes have a dynamic gastric pH, maintaining an acidic environment during digestion of pH 1.5–3 and a more neutral pH when their stomach is empty, around pH 7 (Bessler and Secor 2012). *Caenorhabditis elegans* intestinal pH oscillates between 3.6 and 6 (Chauhan et al. 2013), which is a narrower range than that of other animal species [e.g. 1–7.5 in humans (Brinck et al. 2025); 2–9.5 in flies (Lemaitre and Miguel-Aliaga 2013, Overend et al. 2016)], a potential limitation when studying acidophilic [e.g. *Lactobacilli, Streptococci, Prevotella* spp., and *Helicobacter pylori* (Bik et al. 2006), or alkaliphilic (Thongaram et al. 2003, Liang et al. 2018)] gut bacteria from otherspecies.

At the cellular level, multiple differentiated gut cell types are shared between animals (Ericsson 2019). *Caenorhabditis elegans* simple postmitotic intestine lacks notable conserved cell types including gut stem cells, circulatory and secondary immune cells (McGhee 2007, Dimov and Maduro 2019). Yet, at molecular-level main innate immune processes, proteases, metabolite absorption, lipid metabolism, endocytosis mechanisms, and other major metabolic and signaling pathways are well conserved from worm to human (Miguel-Aliaga et al. 2018, Dimov and Maduro 2019). On balance, *C. elegans* is thus an anatomically, physiologically, and immunologically simpler model devoid of obvious gut compartmentalization, cell renewal or secondary immunity, where conserved molecular interactions between host and gut microbes can be more easily disentangled.

#### Dietary conditions and feeding preferences impact subsequent host–gut microbiota interactions

Host diet directly modulates microbiota composition by modifying the gut microbiota nutritional and biotic (e.g. ingested microbes present on and within foods) environment. This is exemplified by bee species, where divergent genera (*Apis* sp., *Bombus* sp., and *Xylocopa* sp.) can share core microbiota families in similar proportions from a common diet of pollen and nectar (Holley et al. 2022), whereas species with unique diets, such as the oil-collecting bee *Centris decolorata*, exhibit a strikingly different gut microbiota profile (Kardas et al. 2023). In that respect, *C. elegans* diet differs markedly from that of the ectotherm species it is meant to model, and prebiotics and changes to bacterial culture media conditions need considering.

As mentioned earlier, the *C. elegans* pharynx also grinds ingested bacteria very efficiently during development, preventing the establishment of a *bona fide* gut microbiota until adulthood (Dirksen et al. 2020, Zhang et al. 2021; Fig. 3). Depending on the microbe size and hardiness, reaching the worm gut alive may occur at different times for different isolates during the transition from L4 larva to gravid adult (MacNeil and Walhout 2013, Avery Leon and You 2018). Owing to the founder effect (Sprockett et al. 2018), bacteria that reach the worm gut first may get an early advantage for proliferating in the new niche and might dominate gut microbiota composition. Many research groups inoculate *C. elegans* with new microbes from L4 stage (Sowa et al. 2015, Braun and Mor 2025), others do so from adulthood (Garsin et al. 2001b, Sifri et al. 2002, Zárate-Potes et al. 2022) when all bacteria may be equally able to reach the gut alive.

Next, being oviparous, *C. elegans* does not directly pass down its gut microbiota to its progeny. As *C. elegans* then progresses through larval stages in absence of gut microbiota influence, diet during development initially defines its metabolic state until a gut microbiota establishes (Zhou et al. 2019). While defined diets and inactivated bacterial diets mentioned earlier ensure that nutritional differences between individuals and across replicate experiments are kept to a minimum, even isogenic individuals are metabolically distinct, as exemplified by significant differences in health spans and lifespans observed in all worm populations (Stuhr and Curran 2020). Since host–gut microbe interactions work both ways: gut microbes impacting host metabolism and host metabolism impacting gut microbes (Spor et al. 2011, Chang and Kao 2019), individual isogenic worms fed the same diet and then exposed to the same microbial mix, may differ metabolically and thus yield different gut microbiota composition (Berg et al. 2016, Vega and Gore 2017). This effect is more profound when isogenic worms are fed different diets, impacting key metabolic pathways, such as IGF-1/insulin signaling, growth rate to adulthood, or final adult size, which differentially favours gut microbiota isolates (Zhang et al. 2021). Tight control of *C. elegans* diet during development is therefore critical to ensure fair comparisons between test and control worms in gut microbiota studies.

As alluded to earlier, unlike larger organisms for which dead gut microbiotas contribute negligibly to nutrition, for bacterivorous worms, gut bacteria are both dietary and microbiota, making their relative influences as diet versus live microbiota on host physiology challenging to disentangle. Indeed, bacteria as food source yield different fatty acid, amino acid, sugar, and micronutrient compositions with differential impacts on host physiology over the lifespan (Cabreiro and Gems 2013a, Zhang et al. 2017b, Stuhr and Curran 2020). To deconvolve live gut commensal metabolic influences from dead commensal nutritional effects, *C. elegans* fed live microbes are compared to worms fed the same inactivated microbes, assuming that any differences in host metabolism, physiology, or behavior can then be attributed to metabolically active gut microbiota. Such approaches have helped demonstrate processing by gut microbiotas of orally administrated antidiabetic (Metformin) (Cabreiro et al. 2013b), and anticancer (5FU) (Scott et al. 2017a) drugs that could explain differences in efficacy and secondary effects experienced by patients.

Finally, *C. elegans* relies heavily on its sense of smell to profile its habitat and make decisions, including foraging, avoidance, eating, and egg-laying (Katzen et al. 2023). Its behavior is thus modulated by solutes and volatile compounds released by bacteria, affecting their likelihood of being ingested and their opportunity to colonize the *C. elegans* gut. Worms are also capable of learning and modifying their behaviors in response to external stimuli and internal states, notably to develop new preference towards nutritional bacteria or aversion to pathogens from the damage they cause (Kim and Flavell 2020, Flavell et al. 2022). The scent from a pathogenic bacterium alone suffice to elicit *C. elegans* aversive behavior or to trigger entry into the starvation resistant dauer stage (Zhang et al. 2005). Hence, learned associations with previously encountered food or microbial smells may influence how a new bacterium (such as those found associated with other non-colocated ectotherms) is perceived and whether it is ingested (Zhang et al. 2005, Worthy et al. 2018, Velagapudi et al. 2020, Chai et al. 2024), potentially confounding results. To facilitate the study of ectopic gut bacteria from other ectotherms, it may thus be necessary to force the worms to ingest them by covering the full medium surface with the bacterium of interest, as it done for bacterial infection assays (Maadani et al. 2007, Liu J et al. 2024), or precondition worms with the bacterium of interests (Anyanful et al. 2009, Kim and Mylonakis 2012) or defined odorants (Torayama et al. 2007), or mixing ectopic bacteria with their normal food (Chai et al. 2024). Microscopy approaches using fluorescently tagged microbes or traditional colony forming units (CFU) counts can then be employed to confirm actual gut microbiota compositions in individual worms (Dirksen et al. 2020, Kissoyan et al. 2022).

#### Microbiota metabolic compatibility across animals

A general concern in microbiota modeling is whether the bacteria modeled in one organism will be able to produce the same effects in another host. Animals share similar nutritional requirements (Zečić et al. 2019), which are provided by species-specific diet–gut microbiota associations. While their metabolic output might be similar, owing to the diversity of host environments, establishment of ectopic microbiotas in new hosts can be challenging owing to differences in gut niche environments between animal species. Fortunately, the most prominent microbiotas are often cross-compatible at phylum and often down to genus level due to shared metabolic capabilities that match baseline nutritional needs of multicellular eukaryotic hosts (Ley et al. 2008b, Mueller and Sachs 2015). *Escherichia* spp. are a salient example of this as vertebrate gut microbes that provide an excellent monoxenic food source and supportive gut commensal for *C. elegans*, while also being able to support the complex metabolic needs of the kākāpō ground parrot as the dominant genus in its atypical gut microbiota (Waite et al. 2018, West et al. 2023). Another example is that of the *Enterococcus faecalis* probiotic *YM0831*, isolated from a centipede, which can colonize the gut, inhibit intestinal glucose transport and suppress sucrose-induced hyperglycaemia in both silkworms and humans (Matsumoto et al. 2019). As a bacterivorous species dwelling in decaying organic matter, *C. elegans* is exposed to a wide range of bacteria that are not part of its functional microbiota but can colonize its adult gut when ingested. As a ubiquitous species found on six of seven continents (Blaxter and Denver 2012) often transported by larger animals such as gastropods, it has also coevolved alongside land animal species and their microbiotas. Thus far, *C. elegans* has proven amenable to a range of bacterial food sources and gut microbes it does not naturally encounter, including *E. coli, Enterococci, Pseudomonas* spp., *Lactobacilli, Bifidobacteria*, and has proven a useful system to study the human bacterial pathogens *Staphylococci, Streptococci, Salmonella enterica, Serratia marcescens*, and more, indicating wide cross-compatibility with bacterial species of ectotherms and homeotherms despite its different ecology and simpler anatomy (Marsh and May 2012, Clark and Hodgkin 2014).

#### Of protophenotypes and phenologs

Due to important anatomical differences between *C. elegans* and other more complex nonmodel ectotherms, such as arthropods, fish, and amphibians, *C. elegans* diseased or stress phenotypes may not resemble those of other animals. For instance, *C. elegans* is devoid of respiratory and circulatory systems, its neurones are not myelinated, it does not possess an adaptive immune system (unlike all jawed vertebrates), a haematopoietic lineage, bones, or circulatory immune cells, etc (Hobert 2005, Marsh and May 2012, Corsi et al. 2018). Establishing phenotypic correspondences between model and modeled animal are thus essential. In some cases, gross similarities help establishing such equivalencies, defining proto-phenotypes (Dagenhardt et al. 2017, Dwyer 2018, Siaw and Ee-K 2022), in other instances, bioinformatics predictions can be made to identify more distant orthologous phenotypes, called phenologs (McGary et al. 2010, Woods et al. 2013, Golden 2017). Similar approaches may thus need to be employed for studies using *C. elegans* to study nonmodel ectotherm host–gut microbiota interactions.

#### Dealing with differences in immune systems

Nevertheless, host gut immune systems (both innate and adaptive) are shaped by and modulate gut microbiota compositions (Abraham and Medzhitov 2011, Brown et al. 2013, Tomkovich and Jobin 2016). With its lack of adaptive system, *C. elegans* immune responses to and immunomodulation of gut microbiotas will involve conserved innate immune pathways, such as Toll-like, NOD-like, and C-type lectin signaling (Kawasaki and Kawai 2014, Li et al. 2017, Dierking and Pita 2020) and gut–microbiota–brain axis pathways (Nagpal and Cryan 2021, Park et al. 2025), but will not recapitulate exactly other ectotherm’s interactions with the same microbes. While *C. elegans* is a powerful entry-level model to rapidly evidence causal relationships and identify molecular mechanisms of host–gut microbiota interactions, confirmatory experiments must be performed in the host of interest or an experimentally more amenable closely related species. At this stage though, *C. elegans* experiments will have helped refine subsequent experiments and reduce the number of test animals needed (Poh and Stanslas 2024), which is highly desirable when dealing with sentient or endangered species.

### Exploiting *C. elegans* experimental throughput to power the study of nonmodel ectotherm microbiotas dynamics in response to environmental change

To date, *C. elegans* has been used primarily in microbiome research to (1) determine the genetic/metabolic mechanisms driving positive and negative interactions between gut bacteria and host, notably in the context of ageing research (García-González and Walhout 2017), (2) screen for and evaluate potential probiotic interventions meant to promote host health and tackle disease states (Park. et al. 2014). However, this has overwhelmingly been pursued with mammalian health in mind, involving follow-up trials in rodent models, hoping for later human clinical applications (Chen et al. 2021, Rosa et al. 2023). The relative success of such approaches is remarkable considering the differences evoked earlier between *C. elegans* and mammalian physiologies and bodes well for the use of *C. elegans* in the study of ectotherm gut microbiotas, which have been largely overlooked.

Indeed, *C. elegans* use to study ectotherm microbiotas has been patchy. Investigating *C. elegans*’ together with other ectotherms’ microbiotas has helped define the functional profile of the arthropod microbiome (Esposti and Romero 2017). *Caenorhabditis elegans* interactions with microbiotas from marine sediments has also been compared with that of the aqueous nematode *Litoditis marina* highlighting both shared and species-specific natural host–gut microbiota genetic interactions (Xue et al. 2024). Cultivating worms on the simplified native *C. elegans* gut microbiota model CeMbio has also identified protective roles for worm gut commensal isolates against microsporidia infection, a common eukaryotic pathogen also infecting mosquitoes and humans (Jarkass et al. 2024). Whilst limited research has been conducted on nonmodel ectotherm microbiotas, in the original host species or in model organisms, the impact of gut microbes on ectotherm response to temperature has been consistently noted, with specific gut commensals found to confer increased heat tolerance (Moeller et al. 2020, Jaramillo and Castañeda 2021, Dallas et al. 2024, Wu et al. 2025). Some of these effects were shown to be conserved across amphibian hosts, using cross-species microbiota transplantation (Dallas et al. 2024). To date, probiotic interventions have been shown to improve host heat tolerance in flies (Ayyasamy et al. 2021, Jaramillo and Castañeda 2021), worms, aphids, bees, amphibians, lizards, bats, plants, and corals (Fontaine and Kohl 2023, Garcias-Bonet et al. 2024). Thanks to the aforementioned benefits the worm has to offer, using *C. elegans* as recipient host for cross-species microbiota transplants holds promise for identifying the host–gut microbiota molecular mechanisms at play, to design species-adapted probiotic interventions.

A blueprint for ectotherm probiotic screening in *C. elegans* may be derived from existing experimental pipelines that have enabled the development of biological, nematocidal, and insecticidal agents. For instance, the excessive use of commercially available *Bacillus thuringiensis (Bt)—*a soil dwelling bacterium also found within the gut microbiotas of insects, as a bioinsecticide since the 1920’s (Nester et al. 2002) has led to many insects developing *Bt* toxin resistance (Tabashnik et al. 2013), sparking a race for new bioinsecticides. Alongside the co-located roundworm, *Pristionchus pacificus, C. elegans* has been used to screen for new nematocidal *Bacillus* strains from soil, dung, and dung beetles under the assumption that they would also exhibit insecticidal properties (Rae et al. 2010). Identifying a link between insecticidal and nematocidal bacterial strains further led to studying, in *C. elegans*, the repurposing of *B. thuringiensis* strains as potential nematicides against plant parasitic roundworms (Verduzco-Rosas et al. 2021). Other research on the insecticidal toxin complex (Tc) proteins produced by insect gut-commensal bacteria, has exploited *C. elegans* mutants to identify host genes driving nematocidal and insecticidal phenotypes (Sänger et al. 2023). There are also published studies employing *C. elegans* to screen, develop, and test probiotics for homeotherm species, relying on well-established nematode life-trait analyses (lifespan, fecundity, motility, and stress resistance) as readouts for changes in host fitness. These have been applied to mammalian probiotic lactic acid bacteria from human infant faecal samples (Park et al. 2014). Human-targeted probiotics have hence been shown to ameliorate the worm aged-related gut leaking, motility loss, and survival (Ahmadi et al. 2020) and to reduce lipid accumulation, and induce antiinflammatory and neuroprotective effects in *C. elegans* (Goyache et al. 2024). The mammalian commensals *Enterococcus faecium* and *Bifidobacterium lactis* have also been found to extend *C. elegans* lifespan, possibly through the reduction of lipid accumulation, which was used to target lipid accumulation in obese canines subjected to a high-fat diet (Kang et al. 2024). Beyond the identification of probiotic candidates, *C. elegans* have also been used to dissect the mechanisms by which probiotics exert their effects. In one such study, *Lactobacillus fermentum* was found to rely on signaling via a subset of nuclear hormone receptors and the conserved p38 mitogen-activated protein kinase pathway (Park et al. 2018).

In parallel, *C. elegans* has become a convenient experimental platform to support the bioinformatic analysis of host–diet–gut microbe interactions, notably demonstrating how gut microbiota and host metabolic modeling can effectively predict the effects of prebiotics on gut microbiota composition, paving the way for designing pre- and probiotic formulation to address host metabolic issues (Marinos et al. 2024). Thanks to its throughput, the worm model has also been successfully used to characterize tripartite interactions between drugs, host, and gut microbiota. This has been applied to xenobiotics, identifying biotransformations involved in their absorption and bioavailability (Forslund et al. 2015). It has also been applied to the study of prescription drug mechanisms of action and processing by gut microbes (Cabreiro et al. 2013a, Scott et al. 2017b, Pryor et al. 2019). It was notably found that host absorption of Vitamin B6 impacts on the therapeutic potential of fluoropyrimidine 5-FU (Scott et al. 2017a) and render microbiotas resistant to fluoropyrimidines (Rosener 2023). This positions *C. elegans* as an effective tool to accelerate the development of personalized medicines, where the outcomes and efficacies of certain drugs can be predicted and adjusted for different individuals and their gut microbiota make-up (García-González et al. 2017, Haçariz et al. 2021), notably for orally delivered medicines targeting neurodegenerative diseases (Wang and Zheng 2022).

Thus, all the tools and preliminary evidence required are available to make *C. elegans* a powerful experimental host platform to study ectotherm microbiotas and develop microbiota-based interventions that protect ectotherms for the pressures of climate change and anthropic activities. A well-known example of such pressure is neonicotinoids, which at sublethal doses cause chronic impairment of bumblebee natural foraging behavior (Gill and Raine 2014) as well as blunting of *C. elegans* growth, reducing its fecundity, and triggering the degeneration of nicotinic cholinergic neurons (Bradford et al. 2020). Yet, bacterial species related to invertebrate gut microbiotas isolates were found that degrade neonicotinoids in both laboratory and field conditions (Pang et al. 2020), which could form the basis of new protective gut microbiota interventions for affected arthropods. In this scenario, *C. elegans* may be used to (1) screen many related bacterial strains for their ability to degrade neonicotinoids without negatively impacting on *C. elegans* fitness, (2) assess the toxicity of the degradation products, (3) dissect neonicotinoid handling mechanisms employed by host and gut microbiota, and (4) optimize prebiotic and microbial mixes to serve as probiotics for bees. Such approaches may also be expanded to a broader range of environmental isolates to identify other desirable properties when introduced into ectotherm gut microbiotas.

#### A *C. elegans*-based multidisciplinary experimental pipeline to study and identify beneficial gut microbial isolates for nonmodel ectotherm species

**Figure 4:**
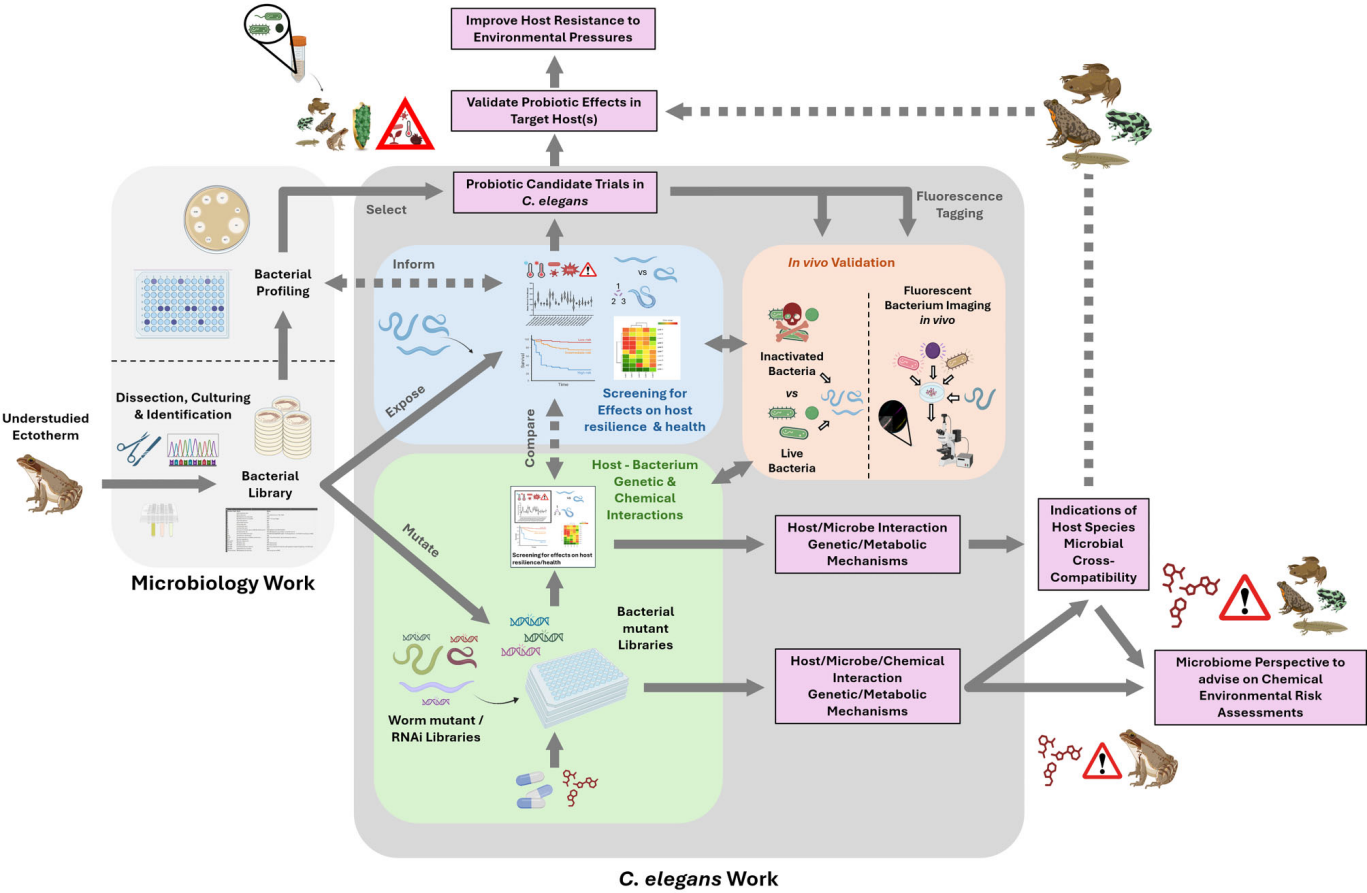
*Caenorhabditis elegans* could be used in a variety of ways in conjunction, building a pipeline to screen and investigate the microbiota of understudied ectotherms and develop products and understanding that can assist in their protection. Illustrated example on an unspecified frog species, created using BioRender and Microsoft Powerpoint.

Whilst utilizing *C. elegans* to study host–gut microbiota interactions of nonmodel ectotherms is interesting in its own right, a systematic, multidisciplinary and *C. elegans*-based experimental pipeline can cost-effectively yield both fundamental insights and remedial strategies to tackle current challenges faced by ectotherms. We propose such an approach here (Fig. 4).

1. Our suggested pipeline would start with identifying common gut microbiota species within the target host, using a sequencing method with high read depth (metagenomics ideally) (Dirksen et al. 2020, Zhang et al. 2021);
2. This would be followed by building a culturable microbial library from healthy host gut content that is as comprehensive as possible. Cultured bacteria may be characterized further using phenotypic microarrays and metabolic profiling by mass-spectrometry approaches (e.g. MALDI-TOFF biotyper) (Khatri et al. 2013, Anani et al. 2019, Mbong Ngwese et al. 2026). This may inform on their metabolic requirements and suitable prebiotics that may support their growth or persistence in a microbial community.
3. *Caenorhabditis elegans* can then be used to screen the cultured microbial isolates, in monoxenic and polyxenic conditions, for beneficial effects on life traits (developmental rate, growth, brood size, behavior, and survival) in the stress conditions of interest (e.g. high temperature, parasites, and pollutants), using available high-throughput *C. elegans* assays (Stroustrup et al. 2013, Olmedo et al. 2015, Koopman et al. 2016, Churgin et al. 2017, Pittman et al. 2017, Benedetto et al. 2019, Haroon and Vermulst 2019, Rahman et al. 2020, Barlow et al. 2022, Bonnard et al. 2022, Gardea et al. 2022, Zavagno et al. 2024, Peng et al. 2025).
4. Microbes of interest may then be investigated further by generating microbial mutant libraries to identify microbial gene mutations that abrogate or enhance *C. elegans* health benefits, and pinpoint beneficial microbial genetic pathways (Khanna et al. 2016, Li et al. 2025a, 2025d).
5. *Caenorhabditis elegans* mutants or worms exposed to gene-specific RNAi may also be screened to identify host genes that mediate the beneficial effects of probiotic microbes, completing the genetic mapping of those beneficial host–gut microbiota relationships (Nasrallah et al. 2023, Peters et al. 2025). Genomic and phylogenetic comparisons across known gut microbes and their hosts may then identify putatively conserved interactions that apply to multiple host–gut microbiota partnerships. This could avoid the need to undergo similarly extensive rounds of screening for sufficiently related ectotherm metaorganisms and instead focus testing on a reduced subset of microbial isolates. For example, research into honeybee protective microbiotas may be predictive of similar interventions in wild bees or in more distantly related invertebrate species, due to naturally occurring similarities in microbiota composition (Kwong and Moran 2016, Zheng et al. 2018).

Yet, to successful and efficiently apply such pipelines to study and harness the potential of currently neglected ectotherms microbiotas, better shared resources are needed, starting with better curated and interoperable microbiota databases, containing well-annotated genomes, consistent metadata on where and when strains were isolated, and their metabolic fingerprints (e.g. MALDI-TOFF spectra). This notably requires enhanced collaboration between instrument manufacturers and academic research groups (Rybnicek and Königsgruber 2019, Loggers et al. 2026) (e.g. to update commercial databases of metabolic spectra as new isolates are sequenced and characterized by researchers). Whilst well-resourced microbiota databases exist for human (Microbiome DB; Oliveira et al. 2018) and other mammals (CramDB; Lei et al. 2022), they are lacking for ectotherms, which hinders new isolate identification and reporting. As the instrumentation needed for optimal workflows if rarely all available in the same institution, funded schemes are needed to access external facilities and to deploy logistics solutions for implementing pipelines across multiple sites (Meder et al. 2016, Strom et al. 2020). Typically, collaborations between academics are setup to achieve that, but institutional and consortium agreements would help narrow the current research and knowledge gap faster, while often improving the usage rate of cutting-edge facilities built at significant expense (Meder et al. 2016, Rybnicek and Königsgruber 2019, Strom et al. 2020).

## Conclusion

Ectotherms gut microbiotas integrate and respond to dietary and other environmental inputs, with impact on their host metabolism, physiology, propension to disease, and stress tolerance. With currently available microbial isolation, sequencing, cultivation, and metabolic modeling methodologies, gut microbiota targeted interventions are highly achievable. However, knowledge on nonmodel ectotherm gut microbial ecologies and metabolism is scarce, despite those organisms representing over 90% of all animal species and being more sensitive to environmental changes than their relatively better studied homeotherm counterparts. This and the inconvenience and expense of rearing nonmodel animals, for which experimental methodologies are lacking, currently limits our ability to close that gap.

*Caenorhabditis elegans* is arguably the best known and characterized ectotherm, owing to its pervasiveness as an experimental animal model, its easy handling, prolificacy, small size, short life cycle and lifespan, transparency, simplicity, and applicability to advanced genetic, biochemistry, and cell biology techniques. It also holds key advantages as a model metaorganism, being easily and inexpensively raised in gnotobiotic conditions, with its adult-only well-characterized and fully cultivatable gut microbiota, genome-wide library of genetic resources, and genetic diversity associated to its worldwide distribution. It is amenable to high-throughput survival, behavioral and *in vivo* microscopy assays, allowing for host physiology and host–gut microbiota dynamic processes to be observed and measured live. Whilst *C. elegans* models are not free from limitations, including internal temperature restrictions and differences in gut morphology, diet, and immune system differences, most derive from its simpler body plan, which also makes its strength for phenomics screening and mechanistic studies as a rapid, affordable and scalable experimental platform.

Recent works utilizing *C. elegans* as a proxy to study other animals’ gut microbiotas and probiotics have mostly favored mammals, owing to their more direct relevance to human health. In doing so, they demonstrated *C. elegans* suitability and power for such purpose. Key conditions are now fulfilled to roll out these approaches to nonmodel ectotherms, and we have provided a roadmap to guide new investigations in this space. Critically, these could afford the development of probiotics for economically and ecologically important ectotherms that boost their resilience to environmental changes brought by global warming and human activities. However, we must highlight the need for collaborations between microbiologists, geneticists, biochemists, and bioinformaticians, and across sectors, the importance of databases and adoption of FAIR principles in data handling, which necessarily involves multisite projects and requires adequate support.
